# Antimicrobial Peptide Databases as the Guiding Resource in New Antimicrobial Agent Identification via Computational Methods

**DOI:** 10.3390/molecules30061318

**Published:** 2025-03-14

**Authors:** Bogdan Marczak, Aleksandra Bocian, Andrzej Łyskowski

**Affiliations:** Faculty of Chemistry, Rzeszów University of Technology, Powstańców Warszawy 6, 35-959 Rzeszów, Poland; 167085@stud.prz.edu.pl (B.M.); bocian@prz.edu.pl (A.B.)

**Keywords:** AMP, AMP databases, diamond alignment, homology modeling

## Abstract

In light of the growing interest in antimicrobial peptides (AMPs) as potential alternatives to traditional antibiotics, proteomic research has increasingly focused on this area. Addressing this significant scientific need, we undertook an initiative to review and analyze the available databases containing information on AMPs. These databases play a pivotal role as a foundation for most AMP-related studies, enabling not only the identification of new compounds, but also a deeper understanding of their properties and therapeutic potential. As part of this study, we evaluated the quality of information within selected AMP databases, considering their accessibility, content, and research potential. The initial step of the analysis involved a comparison of the per-database and cross-database peptide sequences. A *diamond*, high-throughput protein alignment program was used to compare the degree of sequence similarity among peptides across the individual databases. The redundancy of the data was also evaluated. Collected information was used for an in silico evaluation of the selected species’ venom proteomes in order to identify putative antimicrobial peptide candidates. An example candidate was further evaluated via a combination of structural analysis based on the computed homology based structural model, the in silico digestion of the source protein, and the antimicrobial potential.

## 1. Introduction

### 1.1. Antimicrobial Peptides (AMPs)—Natural Defenders with Therapeutic Applications

Antimicrobial peptides constitute a crucial component of innate immunity, and are present in the majority of living organisms. They possess the ability to induce immune responses and exhibit activity against a wide range of microorganisms, including bacteria, fungi, and viruses. Moreover, AMPs can target cancer cells, making them potential therapeutic agents for cancer treatment [1,2].

AMPs are peptide compounds composed of amino acids, typically containing 6 to 100 residues, although peptides with longer sequences also exist. They occur in four main structural forms: α-helices, β-sheets, extended conformations, and loops. Under natural conditions, α-helical and β-sheet structures predominate. Linear peptides lose their structure in solution, whereas cyclic peptides form stable β-sheets maintained by disulfide bonds. Antimicrobial peptides are characterized by a cationic charge and a high content of hydrophobic residues, which contribute to their innate activity against various pathogens and potential applications in therapeutic contexts [1,3]. AMPs are widely distributed in nature and are found in all living organisms. An example of an AMP in humans is the LL-37 peptide, also known as cathelicidin [4]. Other examples of human AMPs include cathelicidins, such as FALL-39 and defensins, which are divided into α-defensins (e.g., HNP-1, HNP-2, HNP-3, and HNP-4, primarily found in neutrophils) and β-defensins (e.g., hBD-1, hBD-2, hBD-3, and hBD-4, expressed in epithelial tissues) [5].

### 1.2. Mechanisms of Action and Immunomodulatory Properties of AMPs

Antimicrobial peptides interact with the cell membranes of microorganisms, leading to their permeabilization and lysis through mechanisms such as the “barrel-stave” model, the toroidal pore model, and the carpet model [6]. They also act at the intracellular level, disrupting key processes such as protein synthesis, nucleic acid synthesis, and cell wall component biosynthesis, ultimately causing structural destabilization and cell death [7].

Furthermore, AMPs exhibit immunomodulatory activity, supporting the recruitment and activation of immune cells and stimulating the adaptive immune response, thereby enhancing their effectiveness in defending against infections [8]. Their application as antibiotics is more efficient and safer, as they are less toxic and do not cause side effects [6].

### 1.3. Classification and Sources of Antimicrobial Peptides

AMPs are classified based on their source, activity, structural characteristics, and amino acid content. Regarding their sources, AMPs are divided into peptides derived from mammals, amphibians, insects, and microorganisms [9].

In mammals, the primary AMP families are cathelicidins and defensins, which protect the organism against infections and exhibit varying expression levels depending on the stage of life, such as in breast milk, which supports infant health [10]. Amphibian-derived peptides, particularly from frogs, play a key role in pathogen defense, with magainin being one of the most well-known examples [11]. In insects, AMPs such as cecropins are synthesized in fat bodies and hemocytes, demonstrating both anti-inflammatory and anticancer activities [12]. Meanwhile, microorganisms, including bacteria and fungi, are sources of AMPs like nisin and gramicidin, which have industrial applications, although their production can be expensive. AMPs possess broad protective properties and potential applications in various fields, including medicine [9].

### 1.4. Antimicrobial Peptide Databases

Antimicrobial peptide databases are a crucial tool in supporting research on their properties, structure, and function. They provide quick access to data such as amino acid sequences, biological sources, physicochemical properties, antibacterial activity, and three-dimensional peptide structures. These data are sourced from the scientific literature, databases like UniProt and NCBI, and experimental results. The database creation process involves collecting, filtering, and verifying information, using tools such as CD-HIT to ensure high data quality [13].

AMPs are classified based on function (e.g., antibacterial, antiviral), structure, or source of origin, facilitating more precise analysis. Some popular databases, such as dbAMP, DBAASP, and DRAMP, offer advanced analytical tools, including machine learning (ML) algorithms. ML models, trained on data from these databases, enable the prediction of peptide activity, the identification of new sequences, and the design of peptides with desired properties, which contributes to combating bacterial resistance to antibiotics. The effectiveness of these models is assessed using metrics like accuracy and specificity, enabling the further optimization and refinement of the research processes. The types of databases are presented in Table 1 [14].

### 1.5. Antimicrobial Peptide Databases—Availability

The selection of AMP databases for analysis was based on the article “A Review on Antimicrobial Peptides Databases and the Computational Tools”. The article presents AMPs as a promising alternative to traditional antibiotics. It includes a review of antimicrobial peptide databases. The databases discussed in the article are presented in Table 2 [14].

### 1.6. Antimicrobial Peptide Databases—Reference Database

The dbAMP is a comprehensive database containing information on antimicrobial peptides, including their sequences, biological activity, post-translational modifications (PTM), structure, and physicochemical properties [15]. In 2024, the database was updated to version 3.0, enriching it with new analytical tools, including a text analysis system based on natural language processing, which enables the automatic identification of publications related to AMPs [16]. This update also introduced extensive data on 53 functional activities of peptides, and information on peptides designed based on proteomic and transcriptomic data [17].

**Figure 1:**
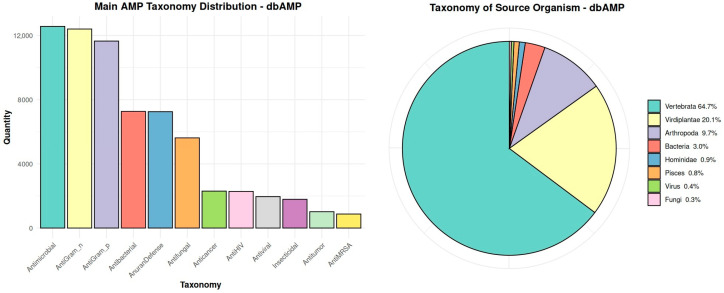
Main taxonomic distribution of antimicrobial peptides and the taxonomic classification of source organisms of these peptides in the dbAMP database.

The dbAMP database was selected as the reference database for the analysis. It stands out from the others primarily due to having the largest number of registered antimicrobial peptides, making it one of the most comprehensive available databases in this field. Regular updates, such as the addition of 9078 AMPs compared to the year 2022, indicate the dynamic growth of this platform. An additional advantage is the precise taxonomy of peptides, which allows for detailed analysis of their diversity, as well as a significant percentage of peptides derived from vertebrates (Figure 1). Thanks to these features, the database is ideally suited to be a reference database.

### 1.7. Antimicrobial Peptide Databases—Review

**Figure 2:**
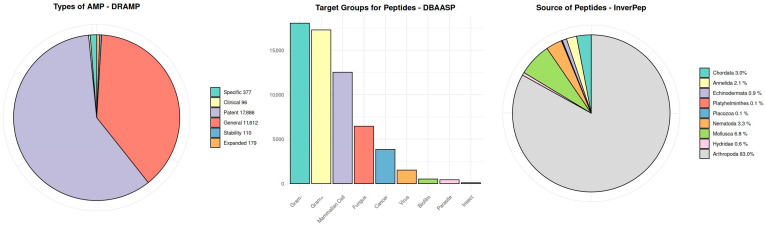
Example charts depicting the properties of datasets from several selected databases.

The selection of the databases listed below for the analysis described in the article is based on their diversity in terms of the number of registered peptides and their types, which allows for a more comprehensive picture of the compared sequences (Figure 2). Each of these databases specializes in different groups of peptides, such as synthetic peptides, antiparasitic peptides, or those exhibiting activity against biofilms, making the comparison of their contents more thorough. As a result, the analysis of these databases facilitates a better understanding of peptide diversity and enables the formulation of broader conclusions.

- APD is one of the first databases on natural antimicrobial peptides, containing information on the sequences and activities of peptides derived from various organisms. Peptides are classified according to their biological properties, such as antibacterial, antiviral, or anticancer activities [18];
- BaAMPs focuses on peptides tested against biofilms, providing verified experimental data [19];
- CAMP is a database that collects data on AMP sequences, their origin, and biological activity, including synthetic AMPs [20];
- CyBase offers data on cyclic proteins, supporting research on their structures and functions [21];
- dadp collects data on antimicrobial peptides, focusing on precursor sequences and their bioactive fragments [22];
- DBAASP collects data on antimicrobial peptides, providing information on their structures, conditions of action, and molecular targets. It also includes predictive tools supporting peptide design [23];
- DRAMP is a database containing peptides with defined sequences, categorized into general and patent sets, with data on toxicity and hemolytic activity [24];
- InverPep focuses on peptides derived from invertebrates, offering data on sequences, structures, and physicochemical properties [25];
- ParaPep specializes in antiprotozoal peptides, offering information on their structures and mechanisms of action [26];
- SATPdb contains data on therapeutic peptides, enabling sequence and structure similarity searches [27].

### 1.8. Diamond–High-Throughput Protein Alignment

Among the various amino acid sequence comparison tools used in bioinformatics analyses, this study utilized *diamond* software as the main tool. It is an advanced open-source tool designed for the rapid alignment of DNA and protein sequences within large databases. *Diamond* delivers a performance significantly superior to that of the traditional methods, such as BLASTX, by employing a simplified protein alphabet and advanced optimization technologies that accelerate the analysis process with minimal loss of precision [28]. The tool enables the efficient processing of large datasets, though it requires substantial computational resources. *Diamond* is particularly valuable in metagenomic projects and the identification of potential antimicrobial peptides (AMPs), though it necessitates users possessing the necessary expertise for the proper configuration and interpretation of results [28,29].

This study aims to evaluate the quality of information within selected AMP databases, considering their accessibility, content, and research potential. Below, we present the results obtained via analysis and a comparison of per-database and cross-database peptide sequences. A *diamond*, high-throughput protein alignment program was used to compare the degree of sequence similarity among peptides across the individual databases. The redundancy of the data was also evaluated.

As a proof of concept, the proposed methodology was used for an in silico evaluation of the selected species proteomes in order to identify putative antimicrobial peptide candidates. An example candidate was further evaluated via a combination of structural analysis based on the computed homology-based structural model, the in silico digestion of the source protein, and the antimicrobial potential.

## 2. Results

During the analysis, in all cases, a greater number of comparisons were obtained than the sum of sequences in the compared databases. This results from the methodology used for sequence comparison. When a reference database, containing, for example, 5000 sequences, is compared with a target database with 3000 sequences, each comparison involves matching each sequence from the reference database with every sequence in the target database.

**Figure 3:**
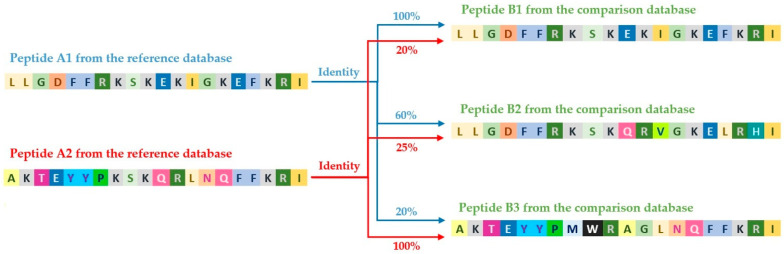
Process of comparing peptide sequences from the reference database and the compared database using the diamond tool.

For example, sequence A1 from the reference database is compared sequentially with all sequences in the target database: A1 with B1 (100% match), A1 with B2 (60% match), and so on. Then, this process is repeated for the next sequence from the reference database, for instance, A2 with B1 (20% match), A2 with B2 (25% match), etc., (Figure 3).

As a result, for each of the 5000 sequences in the reference database, up to 3000 comparisons with the target database are generated. Therefore, the final number of results in the *tsv* file will be equal to the product of the number of sequences in the reference database and in the target database (in this case, up to 15 million possible comparisons, although in practice, only the significant results are often recorded).

Thus, the number of obtained comparisons significantly exceeds the sum of the number of sequences in both databases, as each match between sequences is treated as a separate result (Table 3). The discrepancy between the theoretical and obtained sequence values arises from the use of a reduced alphabet in *diamond* [28], which enhances processing speed but leads to the exclusion of sequences containing non-standard symbols, such as atypical amino acids or errors in the source data.

Based on the conducted research, it is possible to define and standardize indices that describe the repeatability of peptide sequences in databases. The introduced metrics, namely the Database Absolute-Identity Repeatability Index *diamond* (DAIRI_d_) and the Inter-Database Absolute-Identity Repeatability Index *diamond* (IDAIRI_d_), allow for assessing sequence redundancy both within a single database and across multiple databases. The compared value (redundancy) refers to the number of repeating peptide sequences whose similarity reaches 100%, reflecting the percentage of identical peptides within a given database or between databases. These indices were specifically developed to quantify this percentage, based on data obtained through comparisons using the *diamond* tool. The DAIRI_d_ and IDAIRI_d_ metrics provide a standardized approach in order to evaluate how frequently specific peptide sequences reoccur within a given database or across multiple databases.

The results of DAIRI_d_ and IDAIRI_d_ relative to the reference database (dbAMP) are presented in Table 4. Based on the obtained data, it is possible to observe the extent to which the analyzed databases are similar to themselves (self-comparison) as well as to the reference database.

The analysis of the obtained indices from self-comparison and inter-database comparisons with the reference database was extended by an additional assessment of the similarity distribution in relation to peptide sequence length. The graphical representation of the analysis was performed using the *RStudio* environment, which facilitated a more precise presentation and interpretation of the data.

**Figure 4:**
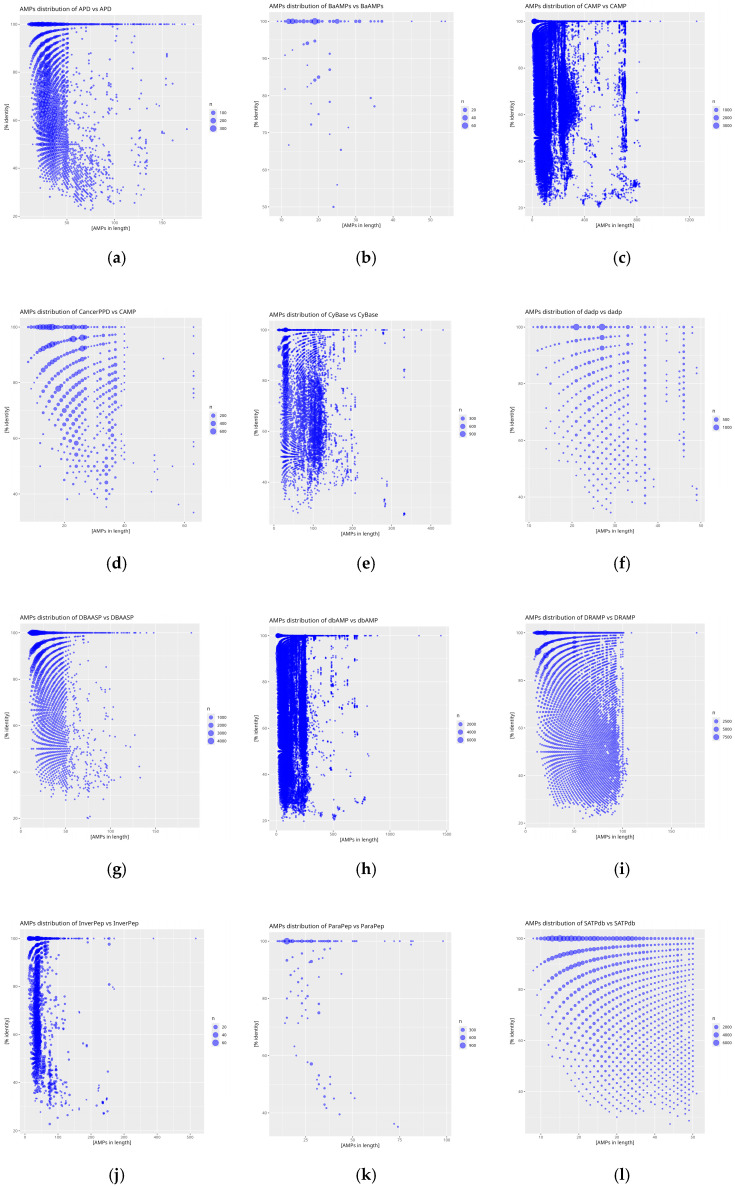
Distribution plots of peptide similarity for a given length for self-comparison of databases: (**a**) APD, (**b**) BaAMPs, (**c**) CAMP, (**d**) CancerPPD, (**e**) CyBase, (**f**) dadp, (**g**) DBAASP, (**h**) dbAMP, (**i**) DRAMP, (**j**) InverPep, (**k**) ParaPep, and (**l**) SATPdb. The *X*-axis represents AMP size in amino acid count and the *Y*-axis % identity is calculated using diamond. The size of the point is proportional to the frequency of the result obtained.

The results of the self-comparison (DAIRI_d_) of the analyzed databases reveal the proportion of peptide sequences exhibiting 100% similarity (Figure 4). The highest percentage of identical sequences is found in the BaAMPs (82.66%) and CancerPPD (70.23%) databases, while the lowest is observed in CyBase (8.99%). The DAIRI_d_ value for the reference database is 17.21%, indicating the relatively low similarity of the database to itself, as this value is less than half of the obtained results and nearly twice as low as the average DAIRI_d_, which is 33.85%.

**Figure 5:**
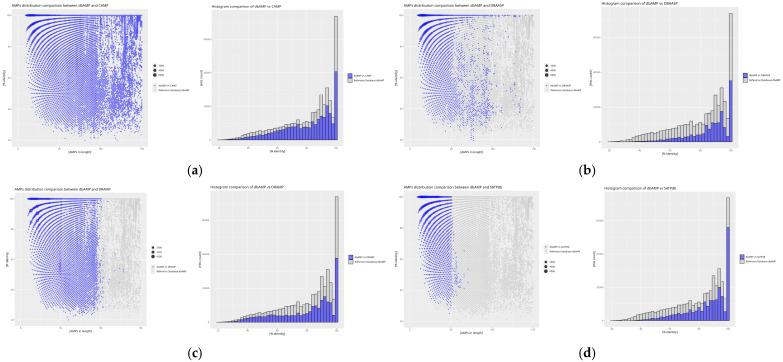
Distribution plots of peptide similarity for a given length for comparison of the reference database with databases of similar sequence counts, as well as histograms for each comparison: (**a**) dbAMP vs. CAMP, (**b**) dbAMP vs. DBAASP, (**c**) dbAMP vs. DRAMP, and (**d**) dbAMP vs. SATPdb. The blue values represent the results of comparing the reference database with a given database, while the gray values originate from the self-comparison of the reference database to highlight differences between the databases. For the scatter plots, the X-axis represents AMP size in amino acid count, the Y-axis % identity is calculated using diamond. The size of the point is proportional to the frequency of the result obtained. For the histogram plots, the X-axis represents % identity as calculated using diamond, the Y-axis ‘hit counts’.

The database Absolute-Identity Repeatability Index *diamond* results are divided into three categories:

Databases with similar sequence counts relative to the reference database dbAMP (Figure 5).

The degree of absolute similarity between the dbAMP and CAMP databases, as determined by IDAIRI_d_ at 27.80%, suggests that more than a quarter of all peptides in both databases share identical sequences. This is represented on the graph by a signal bar at 100% similarity, with minimal scatter for other signals, indicating low peptide diversity in the reference database. A similar result is observed for the DBAASP database, which contains the highest number of identical peptides when compared to the other databases, despite a greater range of values in the dataset. The signal bar at 100% similarity is particularly intense for peptides of up to 25 amino acids in length, showing that this database contains a large number of peptides identical to those in dbAMP, although the length distribution is more varied. In comparison, the DRAMP database shows only 17.12% of identical sequences relative to dbAMP, the lowest value in this comparison, despite a similarly intense signal bar at 100% similarity. The scatter of similarity is small for peptides shorter than 100 amino acids, but increases for longer peptides, suggesting a decrease in the number of similar peptides as the sequence length increases. In the SATPdb database, identical peptides are found only for sequences of up to 50 amino acids in length, with similarity sharply decreasing for longer peptides. This suggests the greater diversity of longer sequences in SATPdb compared to dbAMP as peptide length increases.

**Figure 6:**
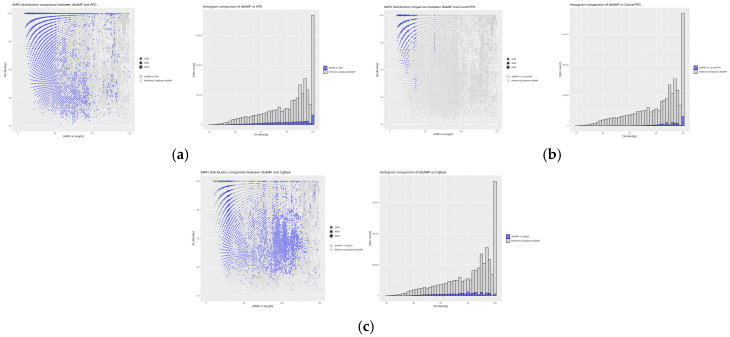
Distribution plots of peptide similarity for a given length for comparison of the reference database with databases of low sequence similarity, as well as histograms for each comparison: (**a**) dbAMP vs. APD, (**b**) dbAMP vs. CancerPPD, and (**c**) dbAMP vs. CyBase. The blue values represent the comparison between the reference database and a given database, while the gray values reflect its self-comparison. The predominant presence of gray values in the plots clearly demonstrates that the reference database is being compared with databases containing significantly fewer sequences than the dbAMP database. For the scatter plots, the X-axis represents AMP size in amino acid count, the Y-axis % identity is calculated using diamond. The size of the point is proportional to the frequency of the result obtained. For the histogram plots, the X-axis represents % identity as calculated using dimond, the Y-axis ‘hit counts’.

Databases with low sequence similarity relative to the reference database dbAMP (Figure 6)–lower IDAIRI_d_ values arise from the normalization of this index. Lower values are characteristic of databases where the ratio of the number of peptides in the compared database to the reference database is low.

In the APD database, which is the least diverse in this comparison relative to dbAMP, 100% similarity is evenly distributed across peptides of up to 50 amino acids in length, with similarity increasing for longer sequences. The CancerPPD database exhibits significant diversity, as similarity is observed only for peptides of up to 50 amino acids long and for a single group of peptides of around 70 amino acids in length. This suggests relatively high sequence diversity in this database compared to dbAMP. On the other hand, the CyBase database, which contains cyclic peptides, shows the lowest percentage of identical sequences relative to dbAMP (0.28%). Despite this, the scatter of data around 100 amino acids indicates low sequence diversity, and the absence of 100% similarity signals for certain peptide lengths suggests the uniqueness of this database.

**Figure 7:**
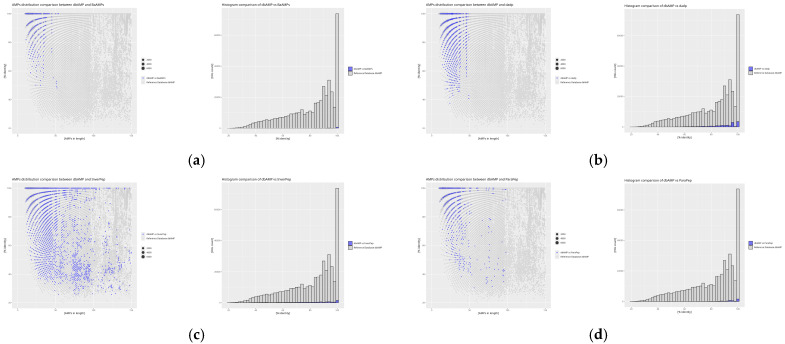
Distribution plots of peptide similarity for a given length for comparison of the reference database with databases with the least similar sequence counts, as well as histograms for each comparison: (**a**) dbAMP vs. BaAMPs, (**b**) dbAMP vs. dadp, (**c**) dbAMP vs. InverPep, and (**d**) dbAMP vs. ParaPep. Similarly to the previous figures, the blue values represent those derived from the comparison of the reference database with a given database, while the gray values correspond to the self-comparison of the reference database. Here, as well, gray values dominate the plots, as the databases compared in this set contain significantly fewer sequences than the reference database. For the scatter plots, the X-axis represents AMP size in amino acid count, the Y-axis % identity is calculated using diamond. The size of the point is proportional to the frequency of the result obtained. For the histogram plots, the X-axis represents % identity as calculated using dimond, the Y-axis ‘hit counts’.

Databases with the least similar sequence counts compared to the reference database (Figure 7)–IDAIRI_d_ decrease to values below 1%.

The BaAMPs database contains the fewest identical sequences when compared to dbAMP. Despite its low IDAIRI_d_ value, the scatter of signals in this database closely matches the signal values of the reference database, indicating low peptide diversity within this database. Similarly, the dadp database, although containing the highest number of identical sequences relative to dbAMP among the databases with the least similarity, shows minimal signal scatter, much like BaAMPs. The InverPep database, despite its low IDAIRI_d_ value, exhibits a scatter of data similar to other databases in this group, suggesting that databases with fewer sequences may rely on more extensive counterparts. Lastly, the results for the ParaPep database align with previous analyses, indicating a similar lack of diversity when compared to the reference database.

In order to demonstrate the applicability of the whole database as a query pattern for the identification of novel, putative AMPs, we have set up an simulation where a selected reference database (dbAMP) is used to assess the potential of a whole proteome. For that purpose, we have selected the proteome of *Naja naja* (*N. naja*, indian cobra). It is one of very few complete venomous snake proteomes available in UniProt resources. Furthermore, it is also one of the limited number of species where the complete genome is also available, opening up possibilities for further experimentation and scientific work [30].

**Figure 8:**
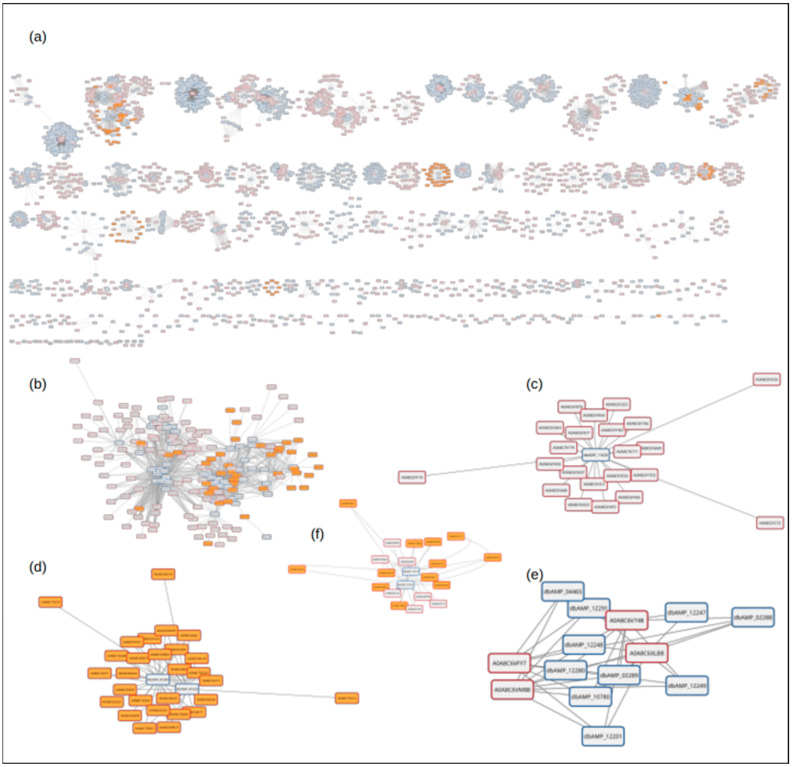
Collection of protein–peptide similarity networks obtained after *N. naja* proteome analysis agains the dbAMP peptide collection. Blue borders indicate dbAMP peptides, red borders *N. naja* proteins, orange-filled boxes represent *N. naja* proteins classified as toxins according to UniProt data. Panel (**a**) represents complete collection of obtained networks, panels (**b**–**f**) present selected examples.

The sequence similarity search of the dbAMP peptide collection against the *N. naja* proteome provided an extensive interaction network consisting of 12,815 records, with the sequence similarity ranging from 100.0 to 19.0%. The complexity of the obtained data file made it impossible to analyze the interaction in the raw format. To interpret the obtained data, they were passed into the Cytoscape software for visualization (Figure 8) [31].

For the proof of concept analysis, we have selected a relatively simple interaction network represented by graph in Figure 8f. In this particular case, sequence similarity matching correlated two AMP peptides dbAMP_07416, 159aa (https://awi.cuhk.edu.cn/dbAMP/information.php?db=dbAMP_07416 (accessed on 17 January 2025)) and dbAMP_10197, 135aa (https://awi.cuhk.edu.cn/dbAMP/information.php?db=dbAMP_10197 (accessed on 17 January 2025)) with total of nineteen proteins from the *N. naja* proteome. The UniProt identifiers, as well as the respective percentage identity toward the AMPs and the beginning and end of the peptide matching sequence, are presented in Table 5.

**Figure 9:**
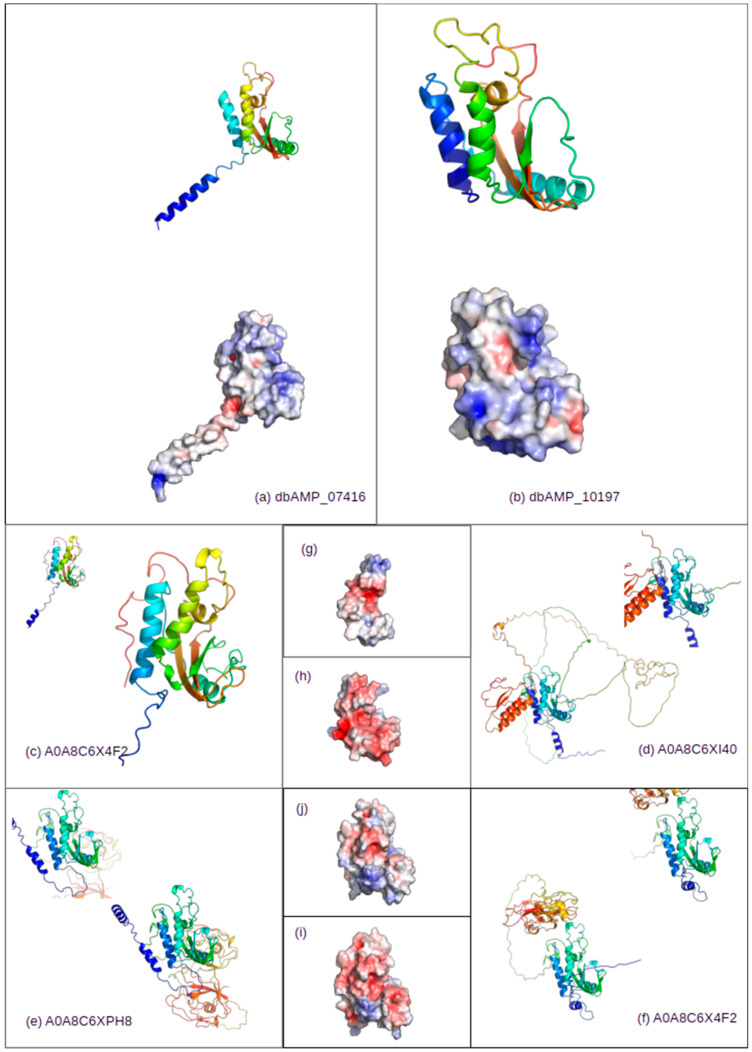
Structural representation of the selected query peptides dbAMP 07,416 (**a**) and 10,197 (**b**) and selected subject proteins A0A8C64F2 (**c**,**g**), A0A8C6XI40 (**d**,**h**), A0A8C6XPH8 (**e**,**i**), and A0A8C6YF13 (**f**,**j**). In the case subject sub-panels, the central model represents whole protein and that localized in the corner sub-structure represents putative AMP domain. Surface representation in all cases represents electrostatic surface potential (red −5.0, blue +5.0), as calculated by PyMOL plug-in APBS electrostatics. All representations were generated using open source PyMOL (v. 3.0.0).

In order to further assess the potential of the identified targets A0A8C64F2, A0A8C6XI40, A0A8C6XPH8, and A0A8C6X4F2, we have analyzed structure corresponding to the related peptides. For all four targets, no experimental models were available in the PDB database, nor in AlphaFold respositories. We have built respective 3D models using the SWISS-MODEL webserver as an automated homology-modeling server. Models were built in the automated mode. Models with highest sequence coverage are analyzed in the manuscript. In each case, a high degree of structural similarity was observed [32]. As an example, Figure 9 presents the combined results for the structural investigation of query peptides, as well as for the four cases indicated in Table 5 in light gray. In the selected cases from the *N. naja* proteome, we wished to demonstrate the presence of the homologous domain in the larger structures. As indicated in Table 5, we have tested selected cases for putative cleavage sites that would allow for the release of the desired domain from the protein. In each tested case, such sites were found either directly flanking the AMP domain or within 10aa from the flanking amino acid. Additionally, for both the query and the putative subject sequences, we have generated electrostatic surface representations.

## 3. Discussion

The results of the self-comparison (DAIRI_d_) reveal that the highest level of redundancy is observed in the BaAMPs (82.66%) and CancerPPD (70.23%) databases, suggesting a large number of repeating sequences. Conversely, the lowest DAIRI_d_ values were recorded for the CyBase (8.99%) and APD (11.70%) databases, indicating a greater uniqueness of the sequences collected within them. The reference database dbAMP exhibits a moderate level of redundancy (17.21%), placing it below the average for all analyzed databases (33.85%).

Inter-database analysis (IDAIRI_d_) reveals that the databases most similar to the reference dbAMP are CAMP (27.80%) and DBAASP (27.92%), which may result from shared data sources or similar sequence selection criteria. Relatively high IDAIRI_d_ values were also obtained for the SATPdb (20.05%) and DRAMP (17.12%) databases. The lowest similarity with dbAMP was recorded for CyBase (0.28%), which contained the relatively fewest sequences similar to the reference database, suggesting that it is the most unique compared to the reference database. In contrast, the DBAASP database exhibits the greatest similarity with dbAMP, making it the most identical of all the compared databases, which may indicate a high sequence homology between these databases.

Analysis of the similarity distribution in relation to sequence length reveals that greater similarity is observed for shorter peptides, while longer sequences exhibit a greater spread of values. In many databases, such as SATPdb, DRAMP, and CancerPPD, identical sequences dominate among peptides shorter than 50 amino acids, with their numbers decreasing as the length increases. Conversely, some databases, such as CyBase, are characterized by greater sequence uniqueness across various length intervals, indicating their specific nature.

The obtained results suggest that the developed indices may serve as valuable tools for assessing the quality and uniqueness of peptide databases. In the future, these indices may be used to optimize bioinformatics analysis and improve the classification and selection of sequences within peptide databases.

In our proof-of-concept analysis, we have demonstrated that the approach similar to high-throughput processing is able to guide the potentially successful identification of new, putative AMP sequences. In our case, we were able to demonstrate that relatively low sequence similarity hits still represent viable targets. All the identified cases retained the key structural aspects of the query peptides (fold, secondary structure domain organization), while varied sequences influenced parameters, potentially improving AMP properties.

## 4. Materials and Methods

### 4.1. Diamond— A Useful Tool in Database Analysis

*Diamond* (v. 2.2.10.164) is an advanced, open-source software designed for matching DNA and protein sequences with large databases such as NCBI-nr and KEGG. It is characterized by exceptional performance—up to 20,000 times faster than BLASTX—allowing for the processing of vast datasets on standard servers, thus eliminating the need for supercomputers in metagenomic and evolutionary analyses [28]. Although this tool requires significant memory and computational resources, for smaller datasets, tools such as BLASTX may be sufficient, making *diamond* particularly optimal for large-scale projects [29].

One of the key factors accelerating *diamond*’s performance is the use of a reduced protein alphabet. The traditional set of 20 amino acids has been replaced with an 11-letter set ([KREDQN], [C], [G], [H], [ILV], [M], [F], [Y], [W], [P], [STA]), enabling faster sequence matching with minimal loss of sensitivity [28]. While this approach may affect precision in cases requiring highly specific matches, the differences are negligible in most applications. Additionally, the use of spaced seed technology allows for the analysis of selected positions in longer sequence fragments, enhancing sensitivity without significantly increasing analysis time. However, for more demanding tasks, such as detecting rare mutations, alternative tools may provide higher accuracy [28,29].

*Diamond* also introduces double indexing, optimizing data locality and reducing the number of memory access operations. These innovations enable the processing of large datasets much faster than tools such as BLAST or MMSeqs2. For instance, in testing, *diamond* processed 246 million reads in just 2.3 hours, whereas BLASTX would require approximately 800,000 processor hours [28,29]. Despite this, such high speeds demand substantial memory resources, which may pose a challenge on less powerful computational machines.

While performance is one of *diamond*’s key strengths, its application in biological analyses is equally important. The tool enables the fast and accurate comparison of protein sequences across various databases, which is crucial for identifying potential antimicrobial peptide (AMP) candidates. *Diamond*’s ability to process large datasets in a short time is particularly valuable in metagenomic projects that require the analysis of hundreds of thousands or millions of sequences. Thus, *diamond* can be used to evaluate the proteomes of various species to identify potential AMP candidates, with results being integrated with structural analysis and antibacterial potential assessments [28,29]. It is important to note that users must possess the necessary expertise to properly configure the tool and interpret the results, especially in more complex analyses requiring parameter adjustments.

### 4.2. Data Preprocessing and Sequence Compatibility for Diamond Tool Analysis

After downloading sequences from the selected database, the analysis proceeded using the *diamond* tool. The first step was to preprocess the data to remove sequences that were not compatible with the *diamond* framework. This program utilizes a reduced alphabet [28], which significantly optimizes processing speed but leads to the rejection of sequences containing non-standard symbols, such as unusual amino acids or characters resulting from errors in the source data. This step is crucial, as it ensures that all analyzed sequences are comprehensible to the tool and can be further processed. Scripts used for data generation are included as a Supplementary Information S4.

### 4.3. Efficient Sequence Comparison with Diamond: Converting Fasta to dmnd and Interpreting Results in tsv

In the next step, the data stored in the *fasta* format were converted into the *dmnd* format. The *dmnd* format is a binary structure optimized for fast homology searching in large databases. Compared to traditional text formats such as *fasta*, *dmnd* is much more efficient in terms of data storage and processing. Its compactness reduces file sizes, which shortens read and write times. Furthermore, this format includes built-in indexes that enable rapid searching and sequence comparison, making it extremely useful in bioinformatics analyses involving large datasets.

**Figure 10:**
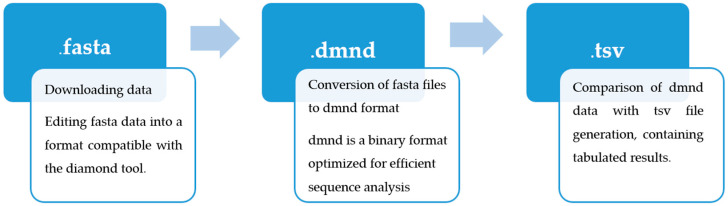
Scheme for performing a comparative analysis of databases using the diamond tool.

After converting the files into the *dmnd* format, the actual comparative analysis was performed. The diamond tool compared sequences from the created database with other datasets, generating results in *tsv* (Tab Separated Values) format. This format, due to its tabular structure, facilitates the later interpretation of the results. The *tsv* file contains key information about sequence matches, such as query sequence identifiers (qseqid) and database sequence identifiers (sseqid), the percentage of identical residues in the match (pident), match length (length), the E value indicating the statistical significance of the match (evalue), and the bit score describing the quality of the match (bitscore) (Figure 10).

### 4.4. Calculation of DAIRI_d_ and IDAIRI_d_ Indices

The Database Absolute-Identity Repeatability Index diamond (DAIRI_d_) represents the percentage of comparisons performed using *diamond* in which 100% sequence identity was observed between databases. It is calculated according to the following formula (Equation (1)).

(1) $$DAIRI_{d}=\frac{n_{diamond\;comparisons\;with\;100\%\;similarity}}{n_{total\;diamond\;comparisons}}\cdot 100\%$$

where *n*_*diamond*_*comparisons*_*with*_100%_*similarity*_ represents the number of comparisons yielding 100% sequence identity and *n*_*total*_*diamond*_*comparisons*_ denotes the total number of *diamond*-based comparisons performed. The resulting value indicates the proportion of comparisons within a given database that correspond to completely identical sequences.

The Inter-Database Absolute-Identity Repeatability Index *diamond* (IDAIRI_d_) extends the concept of DAIRI_d_ by incorporating differences in the number of sequences between the compared and reference databases. It is defined as follows (Equation (2)).

(2) $$IDAIRI_{d}=\frac{n_{diamond\;comparisons\;with\;100\%\;similarity}}{n_{total\;diamond\;comparisons}}\cdot \frac{n_{sequences\;in\;the\;compared\;database}}{n_{sequences\;in\;the\;reference\;database}}\cdot 100\%$$

The first component of this equation represents the proportion of 100% identical comparisons, while the second factor, the ratio of sequence counts in the compared database to those in the reference database, introduces a normalization element that accounts for differences in database sizes. Since databases vary in the number of sequences they contain, direct comparisons may lead to biased interpretations when one database is significantly larger or smaller than another. Even if a smaller database contains all the sequences present in a larger database, their absolute count will be lower, necessitating a normalization adjustment.

The normalization factor ensures that the results are not artificially inflated or diminished due to database size disparities. When both databases contain an equal number of sequences, the normalization factor equals 1, making IDAIRI_d_ equivalent to DAIRI_d_. If the compared database contains fewer sequences than the reference database, the normalization factor is less than 1, reducing the IDAIRI_d_ value and preventing overestimation due to the larger reference dataset. Conversely, when the compared database is larger, the normalization factor exceeds 1, adjusting IDAIRI_d_ to reflect the broader sequence repertoire in the compared dataset.

### 4.5. Graphical Analysis in Rstudio

The data obtained from sequence comparisons between databases, processed using the tool and stored in *tsv* files, were subjected to graphical analysis using the *Rstudio* (v. 2024.12.1) environment. Three types of plots were prepared. The first type consisted of self-comparison scatter plots, which depicted the distribution of peptide similarity (*Y*-axis) in relation to peptide sequence length (*X*-axis). The second type of plots were scatter plots of similarity, identical to the previous type but differing in the data they represented, as they included information on comparisons between the reference database and the other databases. The final type of plots were histograms, illustrating the number of peptides of a given length with a specific degree of similarity.

### 4.6. Naja naja Proteome as an AMP Potential Source Analysis

The proteome of *Naja naja* (*N. naja*, Indian cobra) was downloaded directly from the UniProt database as a plain text *fasta* file [33]. The reference proteome UP000694559 contained 29, 718 entries. After transformation into a *diamomd* database file, it was used during the search, with the complete dbAMP database as a query.

The resulting *tsv* file was then inspected and adjusted for import into Cytoscape (v. 3.10.3), a network analysis and visualization software [31]. In short, the file was imported into a spreadsheet software and modified. Suitable column labels based on the *diamond* nomenclature were introduced. Additionally, helper spreadsheets were prepared based on the data obtained from UniProt. In particular, information allowing for the identification of toxins in the proteome were extracted and formatted according to the Cytoscape requirements. The content of the prepared spreadsheets was imported into Cytoscape in the following sequence: interaction network (calculated similarity between *N. naja* proteins and dbAMP peptides), source and target identifiers allowing for visual differentiation between network elements, identifiers related to the protein type allowing for toxins, and non-toxin diffentiation.

### 4.7. Naja naja Proteome Hits—Structure Analysis and Visualization

Structural models of the *N. naja* targets selected for proof of concept analysis were built using the SWISS-MODEL, a fully automated protein structure homology-modeling server in the automated mode [32].

The predicted structure of the AMP peptides was obtained from the dbAMP database webpage and used without modification.

Electrostatic potential representation was generated using the PyMOL APBS Electrostatics plug-in.

The obtained models were visualized in the PyMOL software (opensource v. 3.0.0) [34].

## Figures and Tables

**Table 1:** AMP database types with descriptions and examples [14].

Database Type	Description	Examples
General databases	They contain various types of AMPs, regardless of peptide family	APD, CAMP, dbAMP
Specific databases	They focus on specific classes of AMPs, such as defensins, cyclotides, or anticancer peptides	CancerPPD, ParaPep
Experimental and predictive databases	They offer both natural and predicted AMPs	CyBase, SATPdb, DBAASP, DRAMP

**Table 2:** Databases described in the article along with their activity status and comparison of the number of AMPs [14].

Database	Status	Number of AMPs (Data from Article 03.2022)	Number of AMPs (Data Current 11.2024)	URL *
APD	Active	1230	5099	https://aps.unmc.edu
BaAMPs	Active **	237	237	https://baamps.it/
CAMP	Active	8160	24,243	https://camp.bicnirrh.res.in
CancerPPD	Active	3490	3491	http://crdd.osdd.net/raghava/cancerppd/index.php
CyBase	Active	1270	1818	https://www.cybase.org.au/index.php
dadp	Active	2571	2571	http://split4.pmfst.hr/dadp/
DBAASP	Active	15,700	22,622	https://dbaasp.org/home
dbAMP	Active	26,440	35,518	https://awi.cuhk.edu.cn/dbAMP/index.php
DRAMP	Active	22,250	30,260	http://dramp.cpu-bioinfor.org/
InverPep	Active	774	774	https://ciencias.medellin.unal.edu.co/gruposdeinvestigacion/prospeccionydisenobiomoleculas/InverPep/public/home_en
ParaPep	Active	863	863	https://webs.iiitd.edu.in/raghava/parapep/home.php
SATPdb	Active	2525	19,192	https://webs.iiitd.edu.in/raghava/satpdb/index.html
ADAM	Inactive	-		
BACTIBASE	Inactive	-		
Defensins Knowledgebase	Inactive	-		
LAMP	Inactive	-		

* All databeses access was verified on 22 February 2025 unless otherwise indicated. Supplementary Information S2. ** The BaAMPs database is inactive as of 22 February 2025, with the last recorded activity in November 2024.

**Table 3:** Comparison of theoretical and obtained sequence counts across databases and number of diamond comparisons with the reference database dbAMP.

Database	Theoretical Number of Sequences	Obtained Number of Sequences	Number of Total *diamond* Comparisons with the Reference Database dbAMP
APD	5099	3167	44,881
BaAMPs	237	221	1504
CAMP	24,243	20,750	269,680
CancerPPD	3491	2849	20,161
CyBase	1818	1757	33,185
dadp	2571	933	18,015
DBAASP	22,622	22,004	22,622
dbAMP	35,518	34,811	419,123
DRAMP	30,260	28,302	211,244
InverPep	774	773	9976
ParaPep	863	513	5053
SATPdb	28,373	25,885	223,633

**Table 4:** Values of the absolute-identity comparison indexes DAIRI_d_ and IDAIRI_d_ in relation to the reference database (dbAMP).

Database	DAIRI_d_ [%]	IDAIRI_d_ Relative to the Reference Database (dbAMP) [%]
APD	11.70	1.75
BaAMPs	82.66	0.29
CAMP	15.00	27.80
CancerPPD	70.23	1.47
CyBase	8.99	0.28
dadp	28.65	0.97
DBAASP	26.12	27.92
dbAMP	17.21	-
DRAMP	24.76	17.12
InverPep	13.20	0.55
ParaPep	73.05	0.30
SATPdb	34.62	20.05

**Table 5:** Results of the *N. naja* vs. dbAMP cross search. The % identity as well as the location of the matching sequence are reported directly, as calculated by *diamond*. The availbility of potential cleavage sites was tested with PeptideCutter (https://web.expasy.org/peptide_cutter/ (accessed on 17 January 2025)) only for proteins with a length significantly longer than the respective peptides (indicted in gray).

UniProt Description	dbAMP _07416		dbAMP _10197		Cleavage Site Available
	% Identity	Seq. Match Start/Stop (Total Length)	% Identity	Seq. Match Start/Stop (Total Length)	
A0A8C6VDU4_NAJNA R3H domain-containing like	32.5	67/219 (253)	32.5	67/219 (253)	
A0A8C6VGD7_NAJNA R3H domain-containing like	32.5	63/215 (249)	32.5	63/215 (249)	
A0A8C6VPF7_NAJNA SCP domain-containing protein	31.9	38/188 (270)	31.9	38/188 (270)	
A0A8C6VPR5_NAJNA SCP domain-containing protein	30.1	38/162 (206)	30.1	38/162 (206)	
A0A8C6 × 1V1_NAJNA R3H domain-containing like	32.5	65/217 (251)	32.5	65/217 (251)	
A0A8C6X4F2_NAJNA SCP domain-containing protein OS = *Naja naja*	36.8	90/173 (302)	36.8	90/173 (302)	yes
A0A8C6X4Z0_NAJNA SCP domain-containing protein	29.8	40/181 (181)	29.8	40/181 (181)	
A0A8C6XI40_NAJNA SCP domain-containing protein	39.2	58/177 (580)	39.2	58/177 (580)	yes
A0A8C6XLY9_NAJNA Peptidase inhibitor 15	32.9	63/223 (258)	33.1	64/223 (258)	
A0A8C6XPH8_NAJNA Cysteine rich secretory protein LCCL domain containing 1	35.0	58/216 (503)	35.0	58/216 (503)	yes
A0A8C6XXU9_NAJNA ShKT domain-containing protein	26.6	26/179 (239)	26.6	30/179 (239)	
A0A8C6XXV5_NAJNA ShKT domain-containing protein	27.2	26/179 (239)	27.3	30/179 (239)	
A0A8C6XZL9_NAJNA ShKT domain-containing protein	26.6	26/179 (239)	27.3	34/179 (239)	
A0A8C6Y1Y2_NAJNA ShKT domain-containing protein	27.8	26/179 (239)	28.7	34/179 (239)	
A0A8C6Y1Z2_NAJNA SCP domain-containing protein	32.4	41/178 (219)	32.4	41/178 (219)	
A0A8C6YF13_NAJNA Cysteine rich secretory protein LCCL domain containing	30.9	55/215 (495)	31.1	56/215 (495)	yes
A0A8C7DRJ6_NAJNA SCP domain-containing	31.0	36/177 (217)	31.0	36/177 (217)	
A0A8C7DRK4_NAJNA GLI pathogenesis related 2 OS = *Naja naja*	30.7	11/145 (154)	30.7	11/145 (154)	
A0A8C7E3S7_NAJNA ShKT domain-containing protein	32.4	41/178 (238)	32.4	41/178 (238)	

## Data Availability

The data presented in this study are available on the respective websites, as described in Table 2, and other publicly available databases, as indicated in publication text.

## Supplementary Materials

The following supporting information can be downloaded at: https://www.mdpi.com/article/10.3390/molecules30061318/s1, S1: Supplementary data file list; S2: Database access status screenshots; S3: *N. naja* proteome analysis data; S4: collection of scrips used for data analysis.

## Abbreviations

The following abbreviations are used in this manuscript:

DAIRI_d_	Database Absolute-Identity Repeatability Index *diamond*
IDAIRI_d_	Inter-Database Absolute-Identity Repeatability Index *diamond*
