# Supplementary material for: Antimicrobial Peptide Databases as the Guiding Resource in New Antimicrobial Agent Identification via Computational Methods
Source: Molecules. 2025 Mar 14;30(6):1318. doi: 10.3390/molecules30061318 (PMC11944441; doi:10.3390/molecules30061318)
Supplement: Supplementary file 1 [file molecules-30-01318-s001.zip › Supplementary/S1_ Supplementary_data_list.docx]

1. S1_ Supplementary_data_list
2. S2_Databases_status_22.02.25
   Database access status list as of 22 Feb 2025.
3. S3_Supplementary_nnaja_dbAMP_analysis_data
   Data used for Cytoscape analysis (generated *tsv* files content imported into Excel):
   1. Sheet nnaja_dbAMP_2
      Results of the *Naja naja* proteome comparison against dbAMP database. Colum labels added according to *dimond* specification.
   2. Sheet nnaja_dbAMP_3
      Filtered out results for the selected example.
   3. Sheet nnaja_toxins_uniprot_2
      Data imported from UniProt with the toxins descriptors separated into columns.
   4. Sheet nnaja_toxin_dbAMP_2
      Results of the *Naja naja* toxins comparison against dbAMP database. Colum labels added according to *dimond* specification.
   5. Sheet nnaja_toxins_id_2
      *Naja naja* extracted toxins descriptors per UniProt ID.
   6. Sheet source_target_id
      *Source* and *target* descriptors for the Cytoscape analysis.
4. S4_Scripts used for data analysis
   1. Folder Data_analysis_scripts contains:
      1. Sample data
      2. Following scripts to clean, convert, run analysis and visualize data:
         clean_fasta.py
         convert_to_dimond.py
         main and run_dimaond_analysis.py
         visualize_rstudio.R
