# Supplementary material for: Antimicrobial Peptide Databases as the Guiding Resource in New Antimicrobial Agent Identification via Computational Methods
Source: Molecules. 2025 Mar 14;30(6):1318. doi: 10.3390/molecules30061318 (PMC11944441; doi:10.3390/molecules30061318)
Supplement: Supplementary file 1 [file molecules-30-01318-s001.zip › Supplementary/S2_Databases_status_22.02.25.pdf]

1. APD – https://aps.unmc.edu

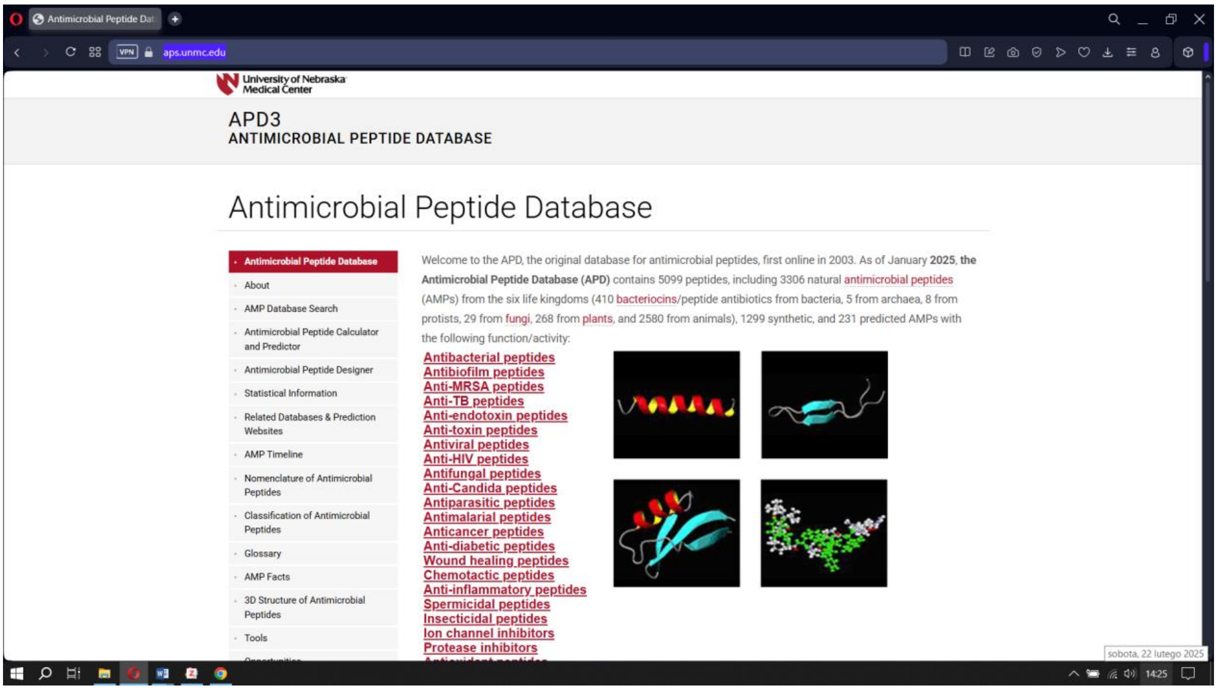

Accessed on 22 February 2025

2. BaAMPs – https://baamps.it

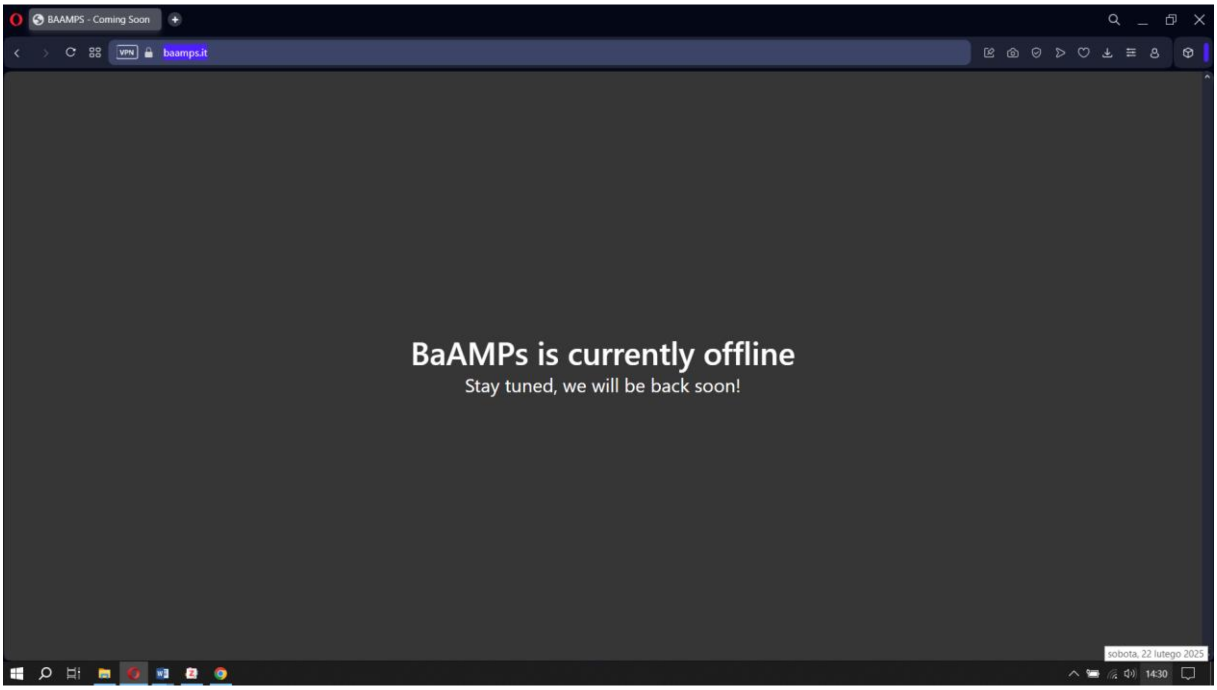

No access on 22 February 2025, last recorded activity in November 2024

3. CAMP – https://camp.bicnirrh.res.in

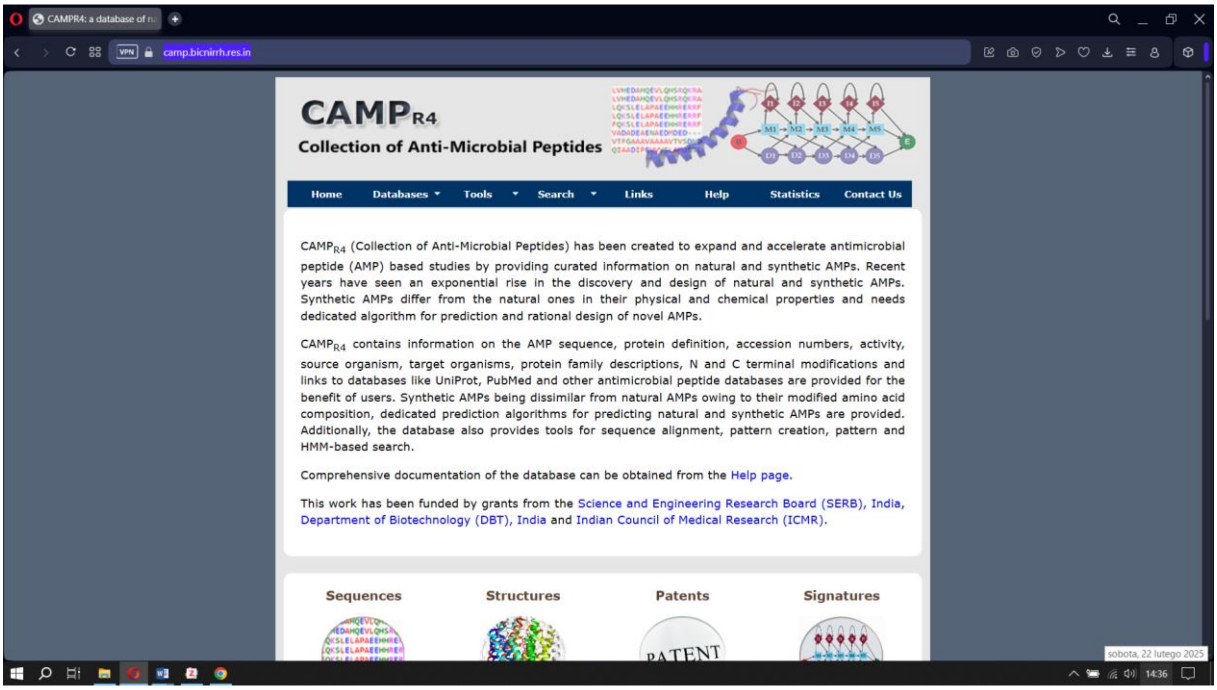

Accessed on 22 February 2025

4. CancerPPD – http://crdd.osdd.net/raghava/cancerppd/index.php

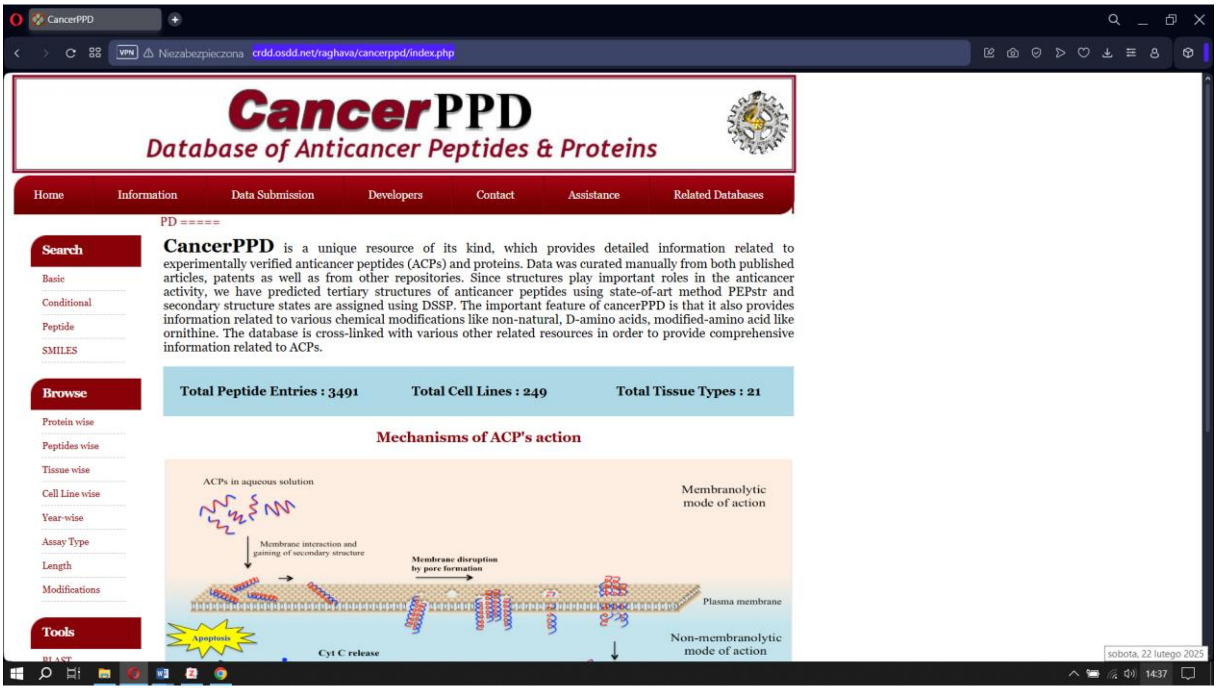

Accessed on 22 February 2025

5. CyBase - https://www.cybase.org.au/index.php

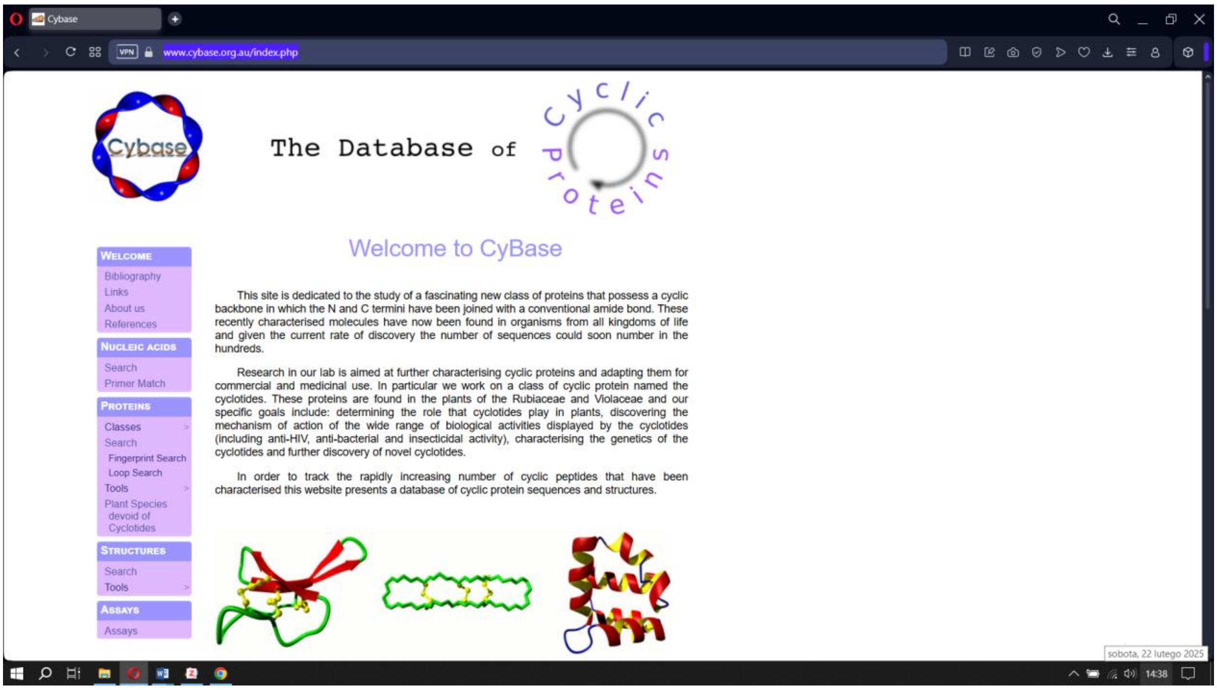

Accessed on 22 February 2025

6. dadp – http://split4.pmfst.hr/dadp/

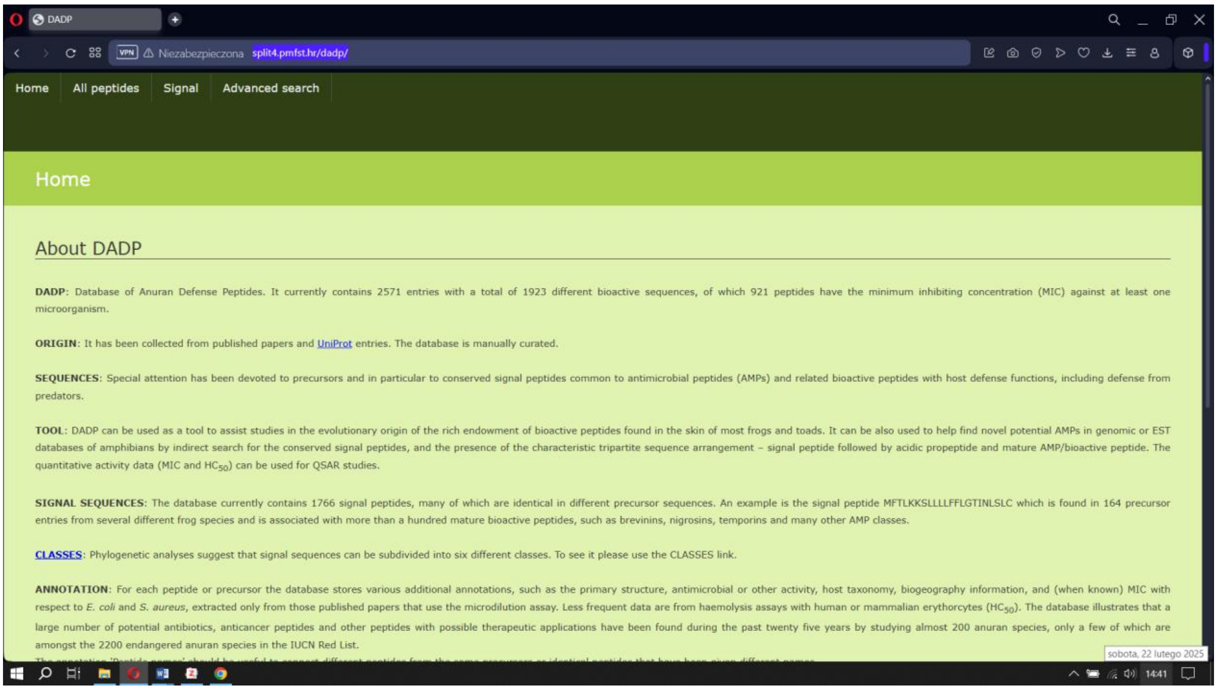

Accessed on 22 February 2025

7. DBBASP – https://dbaasp.org/home

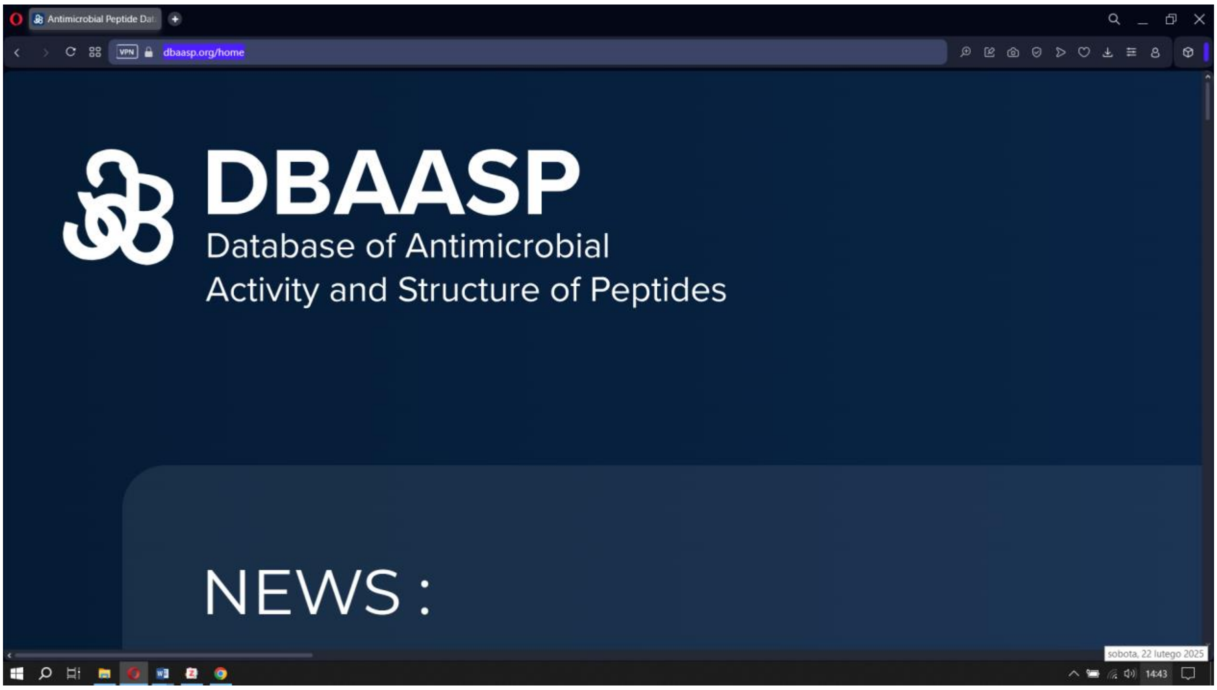

Accessed on 22 February 2025

8. dbAMP – https://awi.cuhk.edu.cn/dbAMP/index.php

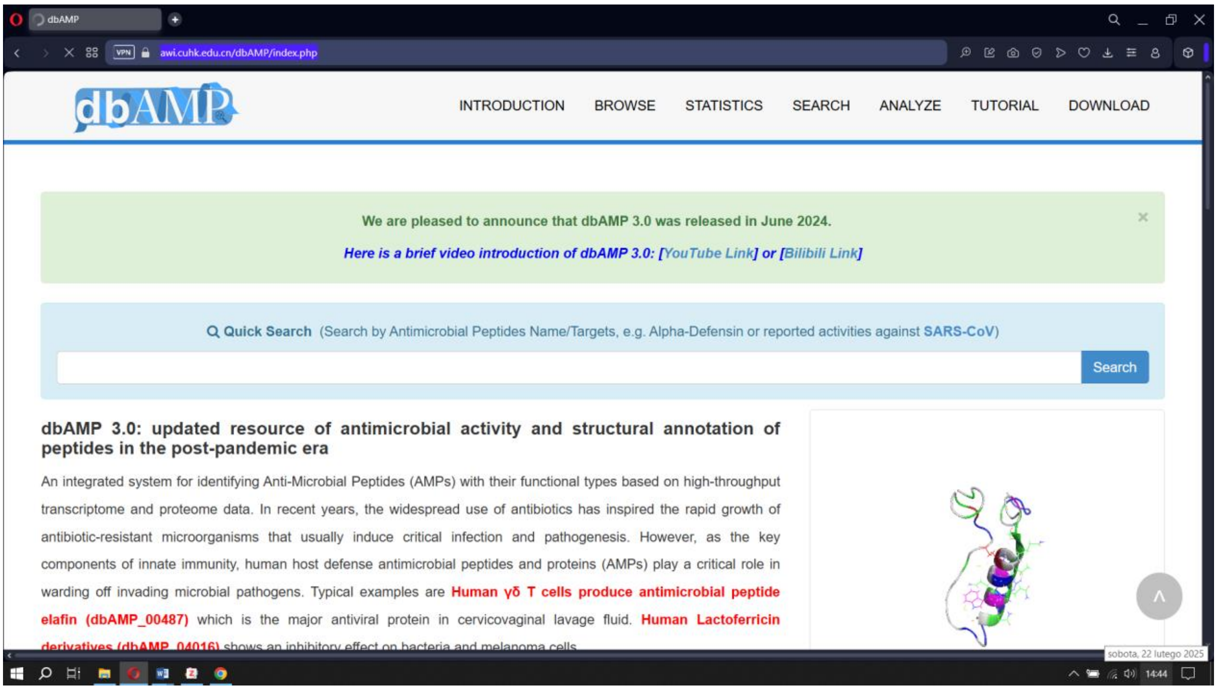

Accessed on 22 February 2025

9. DRAMP – http://dramp.cpu-bioinfor.org/

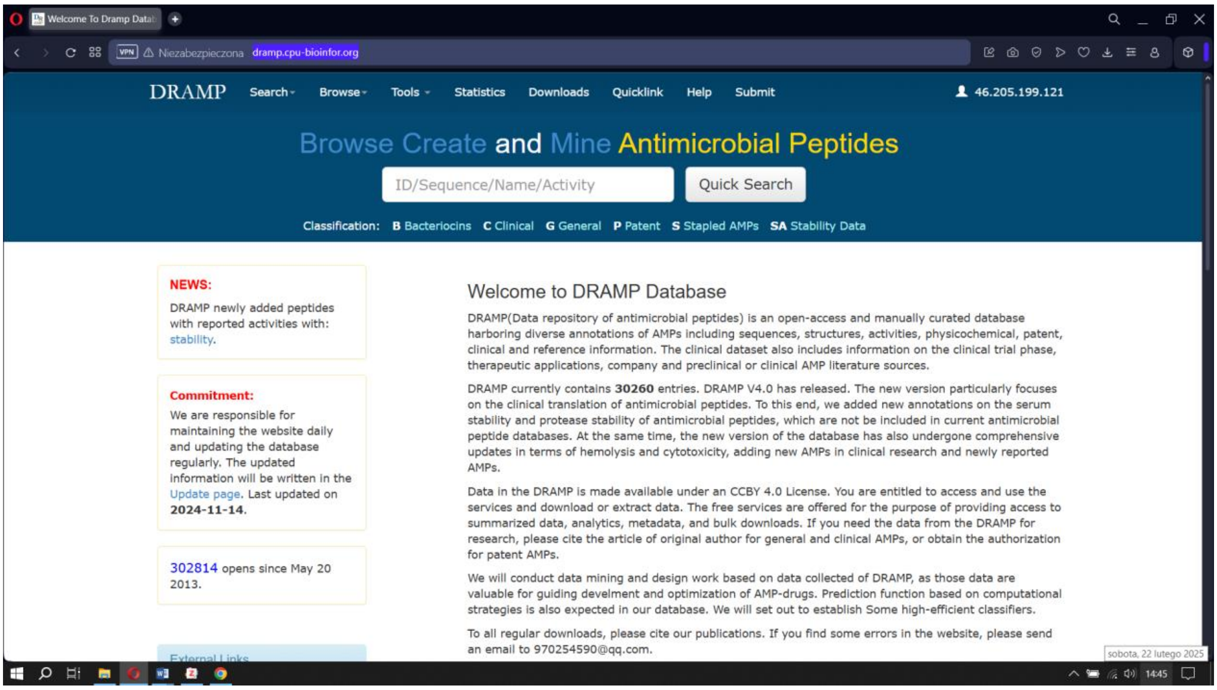

Accessed on 22 February 2025

10. InverPep - https://ciencias.medellin.unal.edu.co/gruposdeinvestigacion/prospeccionydisenobiomoleculas/InverPep/public/home_en

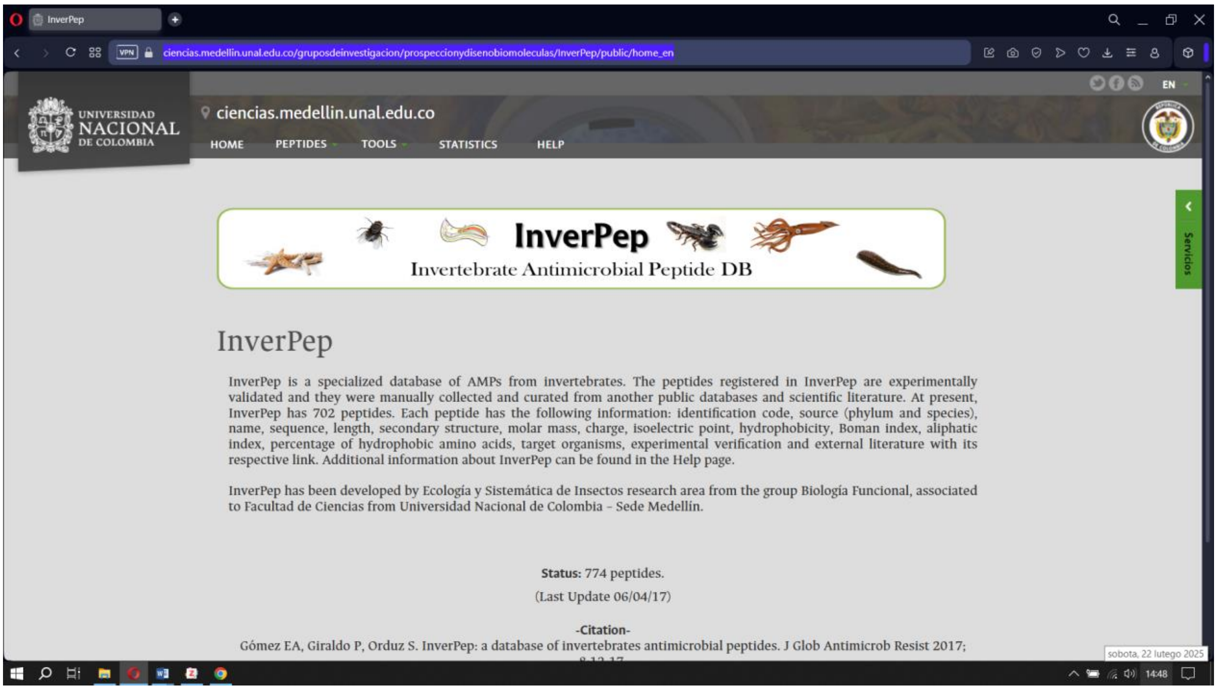

Accessed on 22 February 2025

11. ParaPep – https://webs.iiitd.edu.in/raghava/parapep/home.php

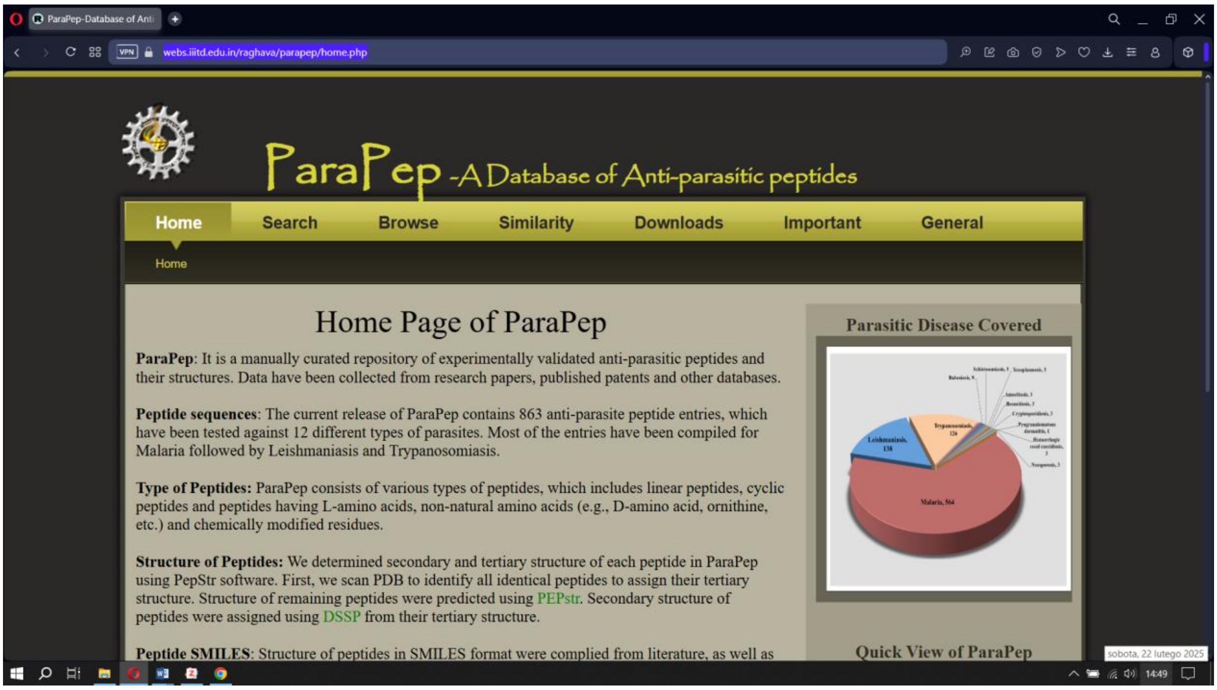

Accessed on 22 February 2025

12. SATPdb – https://webs.iiitd.edu.in/raghava/satpdb/index.html

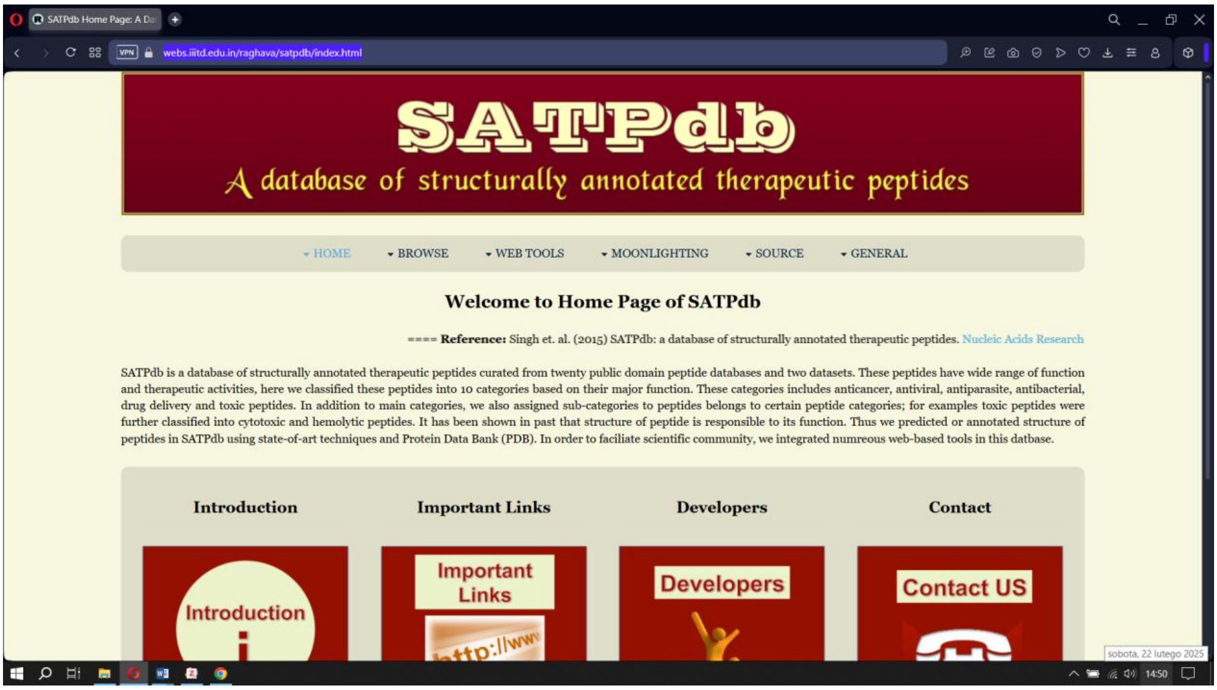

Accessed on 22 February 2025
